# Multiple foci of Rosai–Dorfman disease in colon: a case report

**DOI:** 10.1186/s40792-024-01973-z

**Published:** 2024-07-19

**Authors:** Eri Kisu, Masatsugu Hiraki, Keiichiro Okuyama, Sachiko Maeda, Shin Takesue, Kana Kusaba, Keita Kai, Tatsuya Manabe, Hirokazu Noshiro

**Affiliations:** 1 Department of Surgery, Saga University Faculty of Medicine, 5-1-1 Nabeshima, Saga, 849-8501 Japan; 2 Department of Pathology, Saga University Faculty of Medicine, Saga, Japan; 3 Division of Hematology, Respiratory Medicine and Oncology, Department of Internal Medicine, Faculty of Medicine, Saga University, Saga, Japan

**Keywords:** Rosai-Dorfman disease, Lymphadenopathy, Lymphocytophagocytosis, Emperipolesis, Laparoscopic surgery

## Abstract

**Background:**

Rosai–Dorfman disease (RDD) is an uncommon proliferative histiocytic disorder involving lymph nodes and various organs. Forty-three percent of RDD cases originate from extranodal sites; however, RDD rarely arises from the colon.

**Case presentation:**

A 75-year-old man was admitted to our hospital because of intra-abdominal masses that were incidentally detected during surveillance by computed tomography (CT) after treatment for lung cancer. Enhanced CT showed two mass lesions located in the cecum to the appendix (diameter, 40 mm) and around the sigmoid colon (diameter, 24 mm). Positron emission tomography (PET)-CT revealed an apparent uptake of fluorodeoxyglucose. Intraluminal endoscopy did not reveal definite mucosal abnormalities. These findings suggest the presence of malignant neoplasms including gastrointestinal stromal tumors, lung cancer metastasis, and malignant lymphoma. Exploratory laparoscopy and/or tumor excision were planned to obtain a definitive diagnosis. Based on laparoscopic findings, ileocecal resection and sigmoidectomy were simultaneously performed to excise the tumors. Postoperative histopathological examination revealed multiple RDD originating from the mesocolon side of the cecum and the sigmoid colon. The patient did not receive any adjuvant therapy. No recurrence was observed one year after surgery.

**Conclusion:**

RDD originating from the colon is extremely rare. Tumor extirpation or organ resection is sometimes required to obtain a definitive diagnosis of RDD, and minimally invasive surgery is helpful.

## Introduction

Rosai–Dorfman disease (RDD) is an uncommon proliferative histiocytic disorder associated with sinus histiocytosis and massive lymphadenopathy. However, the etiology remains unclear [1, 2]. RDD involves lymph nodes and various organs, including the bone, skin, soft tissue, central nervous system, eye and orbit, salivary glands, and respiratory tract [2]. RDD involving the digestive tract is rare and occurs in < 1% of extranodal cases [3–5]. Herein, we describe a rare case of multiple foci of RDD in the cecum and the sigmoid colon.

## Case presentation

**Fig. 1:**
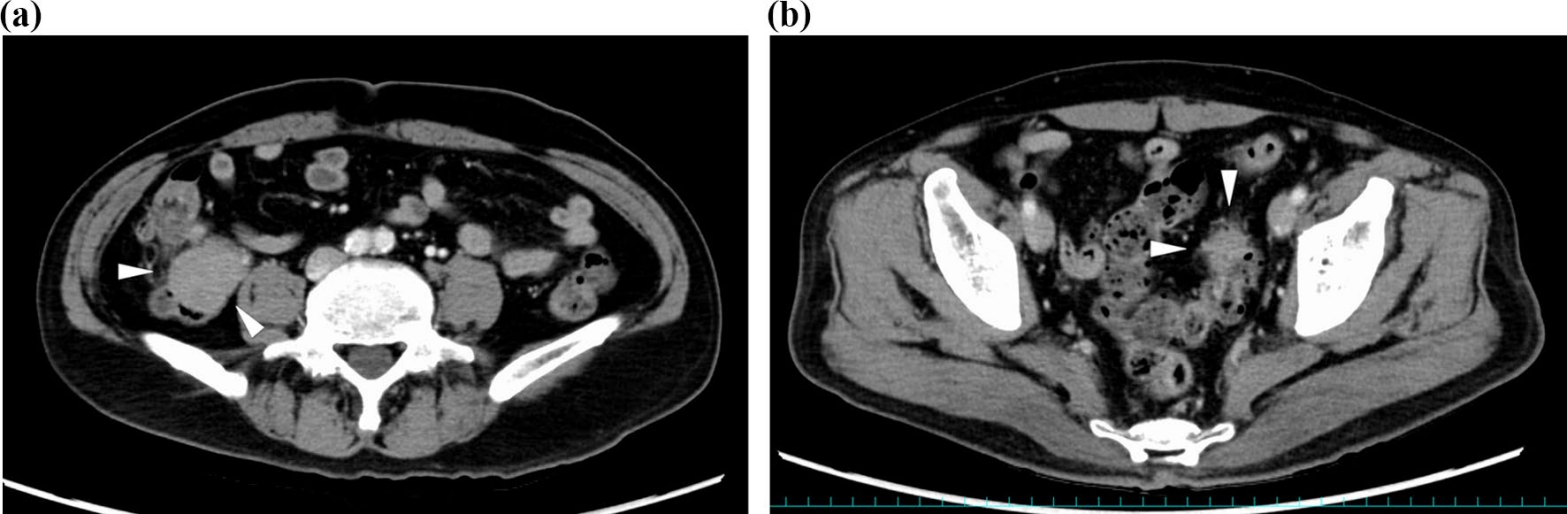
Contrast-enhanced CT showed a tumorous lesion (diameter: 24 mm) at the internal side of the ascending colon with enhancement 40 mm (**a**), and the cephalic side of the sigmoid colon with enhancement (**b**)

**Fig. 2:**
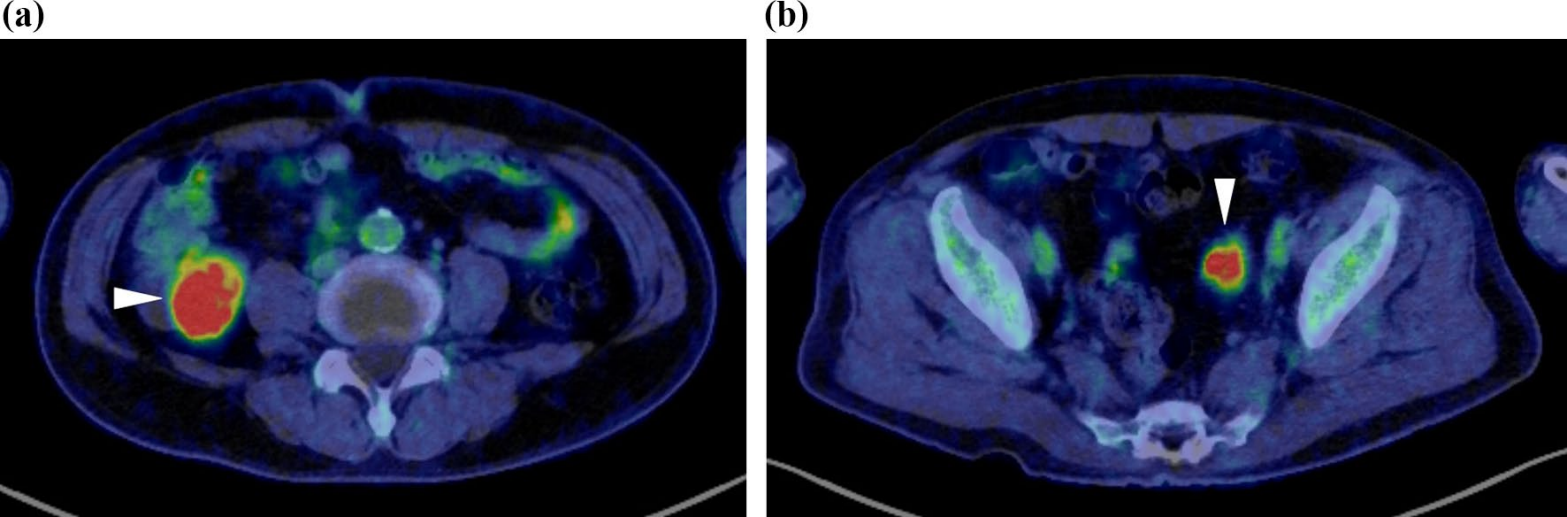
PET-CT showed fluorodeoxyglucose uptake, with an SUV max of 10.682 for the tumor located in the ascending colon (**a**) and 9.509 for the tumor located on the head of the sigmoid colon (**b**)

**Fig. 3:**
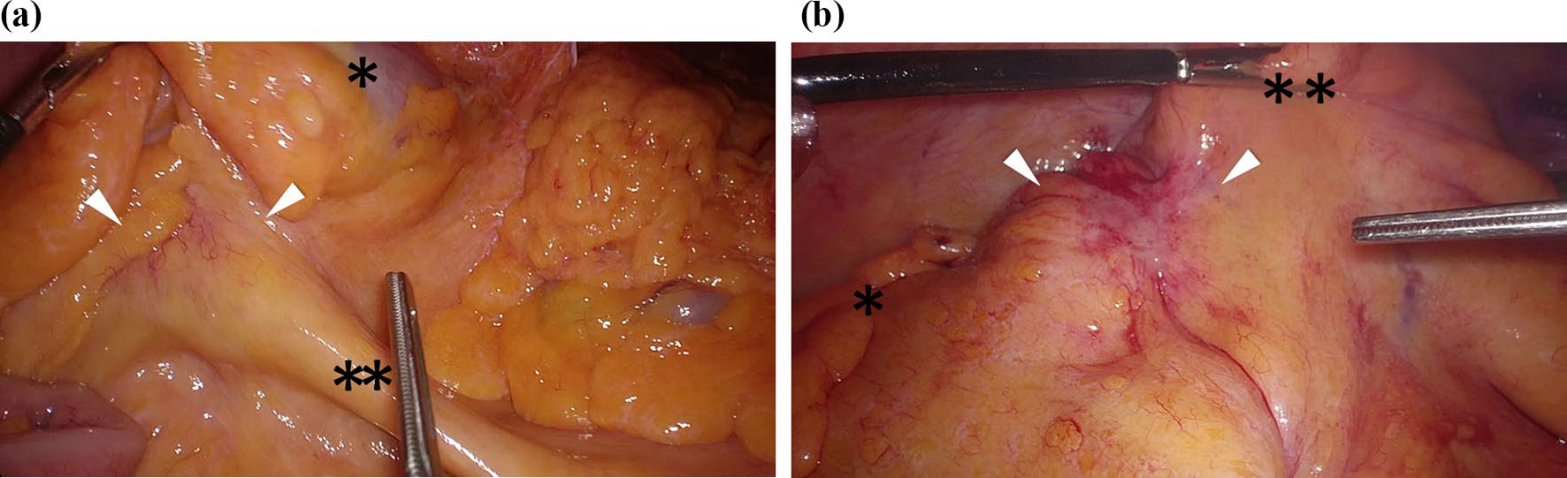
Intraoperative findings. The tumor was observed on the mesocolon side of the cecum (arrowhead, * ascending colon, ** ileocecal vessels) (**a**) and sigmoid colon (arrowhead, * descending colon, ** sigmoid colon) (**b**)

**Fig. 4:**
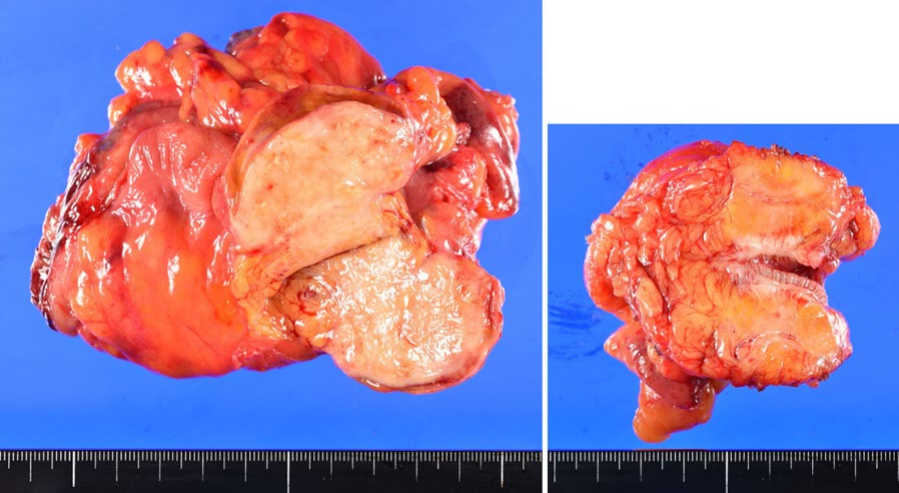
Resected specimen. An yellowish-white mass lesion was seen mainly in the cecum (left) and sigmoid colon (right)

**Fig. 5:**
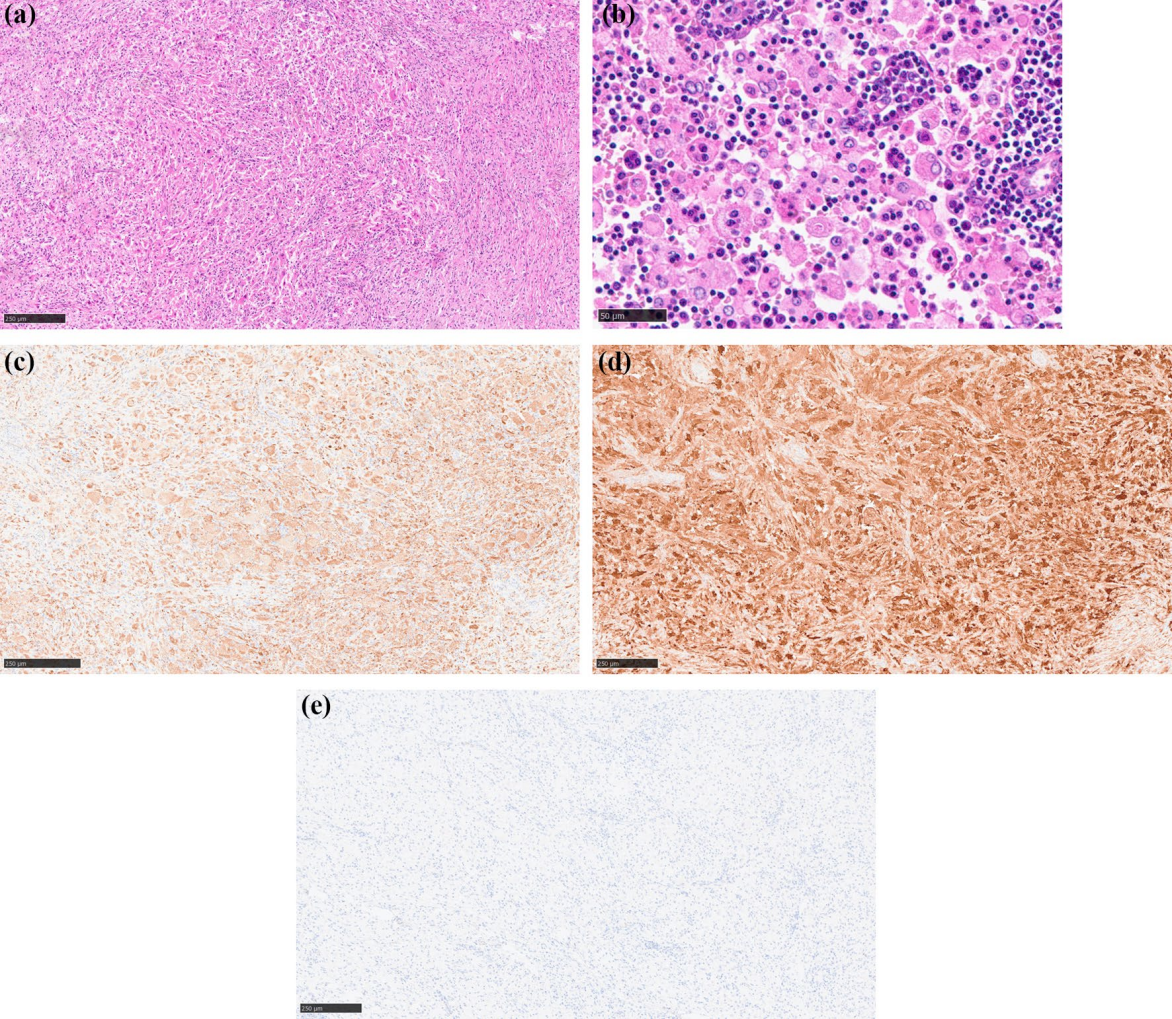
Histology of the colonic mass lesions. A prominent proliferation of spindle-shaped cells with fibrosis and infiltration of histiocytes with pale or acidophilic cytoplasm was observed (**a** HE stain, original magnification: × 100). Many of the infiltrating histiocytes showed emperipolesis. Emperipolesis was especially seen in the lymph node which was included in the resected specimen (**b** HE, × 400). Immunohistochemistry revealed that the infiltrating histiocytes were positive for CD68 (**c** × 100), and S-100 (**d** × 100), and negative for CD1a (**e** × 100)

A 75-year-old man was admitted to our hospital for incidental detection of an intra-abdominal mass on computed tomography (CT) during surveillance after left upper lobectomy for recurrent lung cancer (adenocarcinoma). The sites of lung cancer recurrence were bilateral mediastinal and left hilar lymph nodes. The first-line treatment for recurrent disease was combination chemotherapy with carboplatin and pemetrexed, second-line treatment was nab-paclitaxel monotherapy, and third-line treatment was nivolumab therapy and S-1 therapy. The treatment was changed because of adverse events or disease progression. Subsequently, the patient underwent bronchial artery embolization and radiotherapy for residual mediastinal lymph node metastasis, and partial response was obtained. Thereafter, docetaxel therapy was initiated and continued until the patient’s radiation-induced pneumonitis worsened. However, the recurrent disease disappeared, and the complete response without any treatment lasted for six years. Subsequently, the current event occurred. Enhanced computed tomography (CT) revealed two tumorous lesions located from the cecum to the appendix (diameter: 40 mm) and around the sigmoid colon (diameter: 24 mm). Both tumors showed contrast-medium enhancement (Fig. 1a, b). Positron emission tomography (PET)-CT revealed fluorodeoxyglucose uptake, with maximum standardized uptake values (SUV) of 10.7 and 9.5, respectively (Fig. 2a, b). The lesions showed isointensity on T1-weighted MRI, mildly high intensity on T2-weighted MRI, and high intensity and low apparent diffusion coefficient (ADC) values on diffusion-weighted MRI. Lower gastrointestinal endoscopy revealed no mucosal lesions or submucosal tumors on biopsy or fine-needle cytology examination. A CT-guided biopsy was not performed because of the increased risk of dissemination to the adjacent intestine. The levels of tumor markers were also measured. CA19-9 level was slightly elevated (68 U/ml), while his soluble interleukin-2 receptor level was within normal limits (442 U/ml). Based on the above findings, gastrointestinal stromal tumors of the colon and mesentery, recurrence of lung cancer, and malignant lymphoma were considered differential diagnoses. Although a malignant neoplasm was strongly suspected, a definitive diagnosis could not be made. Therefore, exploratory laparoscopy and/or tumor excision were planned. Five ports were placed, and pneumoperitoneum was performed. Laparoscopic findings showed that both tumors originated on the mesocolon sides of the cecum (Fig. 3a) and sigmoid colon (Fig. 3b). Therefore, laparoscopic ileocecal resection and partial resection of the sigmoid colon were performed. For both lesions, partial resection was performed with sufficient resection margins without lymph node dissection. Anastomosis was performed using an intracorporeal functional end-to-end anastomosis. During surgery, rapid intraoperative histology was performed for both lesions after resection. However, a definitive diagnosis was difficult to make. The operation time was 4 h and 7 min, and the blood loss was 37 ml. The 2 tumors were similar in appearance and consistency. Further macroscopic examination of the excised specimens showed that the tumors could not be separated from the colon walls, and the cut surfaces of the tumors were yellowish-white and uniformly solid (Fig. 4). Histological examination of the main tumor demonstrated prominent proliferation of spindle-shaped cells with fibrosis and infiltration of histiocytes with pale acidophilic cytoplasm (Fig. 5a). Histiocytes showed eosinophilia, foamy sporulation, and irregular shape. They had lymphocytes and other cells with internal halos and emperipolesis was confirmed. Emperipolesis was particularly observed in the lymph node, which was included in the resected specimen (Fig. 5b). Immunostaining showed that the histiocytes were positive for CD68 and S-100 and negative for CD1a (Fig. 5c–e). Only a few IGg4-positive plasma cells were observed in this study. Therefore, the patient was diagnosed with RDD arising from the cecum and sigmoid colon. No postoperative complications occurred, and the patient was discharged from the hospital on postoperative day 11. One year after surgery, no recurrence was observed.

## Discussion

Here, we describe the case of a patient with multiple foci of RDD in the cecum and sigmoid colon. RDD is a rare non-Langerhans cell histiocytosis first reported in 1965 by Destombes [6]. Later, Rosai and Dorfman summarized 34 cases and coined the term sinus histiocytosis with massive lymphadenopathy, which was later changed to RDD [6]. Histologically, the hallmark of RDD is the presence of variable numbers of intact lymphocytes within the histiocytic cytoplasm, a phenomenon referred to as lymphocytophagocytosis or emperipolesis, which is defined as lymphocytic penetration of and movement into the histiocyte [4]. Immunohistochemical studies are widely accepted for diagnosis, and the typical histiocytic markers used for diagnosis demonstrate positivity for S100, fascin, and/or CD68 [3]. Reactive inflammatory processes such as immune deficiency and viral infection have also been suggested to play a role in the pathogenesis of RDD, although no definitive evidence to support any etiology has been found [7].

Classic RDD presents with massive bilateral painless cervical lymphadenopathy with associated fever, weight loss, and night sweats [8]. Most patients with RDD with nodal involvement present with cervical, mediastinal, axillary, and inguinal nodes [9]. However, our patient was asymptomatic, had no massive lymphadenopathy, and had an extranodal lesion in the colon. The main lesion originated from the colon, and the lymph nodes which was included in the resected specimen also contained RDD. It has been reported that 43% of patients with RDD present with extranodal involvement, including the skin, soft tissues, upper and lower respiratory tract, bone, oral cavity, and genitourinary tract [3, 5, 9]. However, RDD involving the colon is a rare condition. A search of the PubMed database using the keywords “Rosai–Dorfman disease” and “colon” for articles published from 1981 to December 2023 and related articles identified 21 cases of RDD of the colon in 13 articles, including the current case [2, 7, 10–20]. Owing to the paucity of information, it was difficult to discuss the actual cause of the occurrence and progression, of RDD from the 21 previously reported cases. However, we speculated that one of the possible causes of this case might be the long-term chemotherapy that our patient underwent for lung cancer. Shukla et al. reported a case of relapse after treatment for Hodgkin disease. Recurrent lesions in the cervical lymph nodes and bone marrow responded well to chemotherapy. However, only the ileocecal mass was ineffective and resected, and a diagnosis of RDD was made [13]. Although a report of only 2 cases does not indicate an increased risk of developing RDD after chemotherapy, other neoplastic lesions, including RDD, might be considered if the recurrent lesions do not respond to treatment as expected or if they recur atypically.

A definite diagnosis of RDD in some organs cannot always be made before surgery because sufficient tissue specimens cannot be obtained. No standard therapy has been established for RDD treatment. However, surgical resection might be helpful for both the diagnosis and treatment, while minimally invasive surgery would be helpful. Among the previous cases, none of these reports described multiple RDD involving the colon treated with minimally invasive surgery, and no reports of laparoscopic surgery for primary RDD of the colon other than our case. In our case, rapid intraoperative histology was performed for both colonic lesions after the resection. However, it was still difficult to make a definitive diagnosis. Even if no malignant findings were found by rapid intraoperative histology, PET-CT still showed the uptake of fluorodeoxyglucose in both lesions, and malignancy was initially suspected. Thus, surgical resection was considered to be necessary.

In one case, multiple tumors occurred simultaneously in the colon, as in this case [20]. As mentioned in the Introduction, primary RDD of the gastrointestinal tract is rare, and classic RDD is characterized by bilateral lymphadenopathy [8]. Therefore, multiple lesions may be uncommon in RDD.

Regarding the prognosis of RDD, recurrence was observed in 2 of the previous 21 cases [14, 15], 1 of which died due to recurrence [15]. Due to the small number of previous cases of RDD originating from the colon, it would be difficult to determine the pattern of recurrence or recurrence rate. However, regular follow-up should be considered even after complete resection.

## Conclusion

We encountered a rare case of multiple foci of RDD involving the cecum and sigmoid mesocolon that was successfully treated using minimally invasive surgery.

## Data Availability

All data supporting the conclusions of this article are included within the published article.

## Abbreviations

RDD: Rosai–Dorfman disease
CT: Computed tomography
PD: Progressive disease
PET: Positron emission tomography
SUV: Standardized uptake value
MRI: Magnetic resonance image
ADC: Apparent diffusion coefficient
